# A multicenter, prospective study evaluating the impact of the clinical pharmacist-physician counselling on warfarin therapy management in Lebanon

**DOI:** 10.1186/s12913-018-2874-7

**Published:** 2018-02-01

**Authors:** Nermine S. Choumane, Diana N. Malaeb, Bassem Malaeb, Souheil Hallit

**Affiliations:** 1 Lebanese International University, School of Pharmacy, Beirut, Lebanon; 2 American University of Beirut Medical Center, Beirut, Lebanon; 3 Saint-Joseph University, Faculty of Pharmacy, Beirut, Lebanon; 4 Holy Spirit University, Faculty of Medicine and Medical Sciences, Kaslik, Lebanon; 5 Psychiatric Hospital of the Cross, Research Department, P.O. Box 60096, Jal Eddib, Lebanon; 6 Occupational Health Environment Research Team, U1219 BPH Bordeaux Population Health Research Center, Inserm - Université de Bordeaux, Bordeaux, France; 7 Biakout, Lebanon; 8 INSPECT-LB: Institut National de Sante Publique, Epidemiologie Clinique et Toxicologie, Faculty of Public Health, Lebanese University, Beirut, Lebanon

**Keywords:** Clinical pharmacist, Counselling, Warfarin, Patient knowledge, Therapeutic INR

## Abstract

**Background:**

Health care professionals (HCP) are known key elements of effective patient’s counselling and education. For patients taking warfarin, education about the dose, side effects, and toxicity is clearly identified as a cornerstone of achieving improved health and quality of life. The study objective was to evaluate the patients’ knowledge about warfarin and assess the impact of the health care professionals’ counselling in enhancing patients’ knowledge in achieving warfarin therapeutic outcomes.

**Method:**

A six-month prospective multicentered study was conducted in three hospitals, enrolling 300 patients admitted to the cardiac care unit and internal medicine departments. Patients’ warfarin knowledge and INR levels were assessed before and after the clinical pharmacist counselling. The main therapeutic outcome was the impact of the clinical pharmacist-physician counselling on improving patient’s education and achieving therapeutic INR level.

**Results:**

A higher mean knowledge about warfarin score was found after counselling as compared to before counselling (4.82 vs 13.2; *p* < 0.001). Likewise, the drug dose (1.05 vs 1.88), drug toxicity (0.41 vs 1.92), drug-drug and food-drug interactions (0.02 vs 1.89), therapeutic INR and general drug knowledge scores (2.66 vs 4.68) were significantly higher after as compared to before counselling (*p* < 0.001 for all variables). The percentages of patients who achieved therapeutic INR levels pre/post counselling was 37.2% and 74.4% respectively (*p* < 0.001).

**Conclusion:**

Based on the study findings, HCP play a major role in enhancing patients’ knowledge about the factors that affect warfarin therapeutic outcomes. This study highlights the need to establish and develop strategies for appropriate warfarin utilization in Lebanon.

**Electronic supplementary material:**

The online version of this article (10.1186/s12913-018-2874-7) contains supplementary material, which is available to authorized users.

## Background

Vitamin K antagonists, namely warfarin, remain one of the main oral anticoagulant treatments used for the prevention and treatment of cardiac, thromboembolic and hypercoagulable diseases [1–4]. Because of its narrow therapeutic index, warfarin requires regular monitoring and dose corrections to retain its optimum anticoagulation effect [3]. Monitoring the International Normalized Ratio (INR) is mandatory to optimize patient outcomes and minimize the risk of thrombosis, without increasing the risk of bleeding complications [5–7]. In fact, previous studies revealed that warfarin adverse reactions were responsible for considerable hospital admissions [8, 9]. Indeed, warfarin is listed among the top ten drugs to cause the largest number of serious adverse events reported during the last two decades, according to the adverse events reporting system of the Food and Drug Administration [3]. Achievement of therapeutic outcomes in patients maintained on warfarin is hindered because it is associated with significant inter-patient variability related to age, gender, ethnicity, body weight and genetic variations, and a wide range of drug interactions [10, 11].

A useful strategy that can be used to decrease possible adverse drug reactions is to provide patients with adequate education in order to improve their knowledge. This would also allow patients control their INR more, and consequently decrease the frequency of checking it and the hospitalization rate [12–14].

Previously conducted studies reported that warfarin knowledge among patients is often inadequate [15–17], while others showed an improvement in anticoagulation control in patients provided with the appropriate information [18, 19]. Therefore, this study was conducted to evaluate the patients’ knowledge about warfarin and assess the impact of the health care professionals counselling in enhancing patient’s knowledge in achieving warfarin therapeutic outcomes.

## Methods

### Setting

A cross-sectional population-based prospective multicentered study was conducted between January and June 2016. Patients were recruited from three urban Lebanese university hospitals chosen randomly from the list of hospitals provided from the Lebanese Order of Pharmacists. The questionnaire was administered face-to-face by trained researchers, who had a training prior to the start of the data collection to ensure the quality of research. Participants who were on warfarin for any of its approved indications (venous thromboembolism (VTE), atrial fibrillation (A.Fib), valve disease/replacement, stroke or Systolic Left Ventricular Dysfunction (SLVD) were enrolled in this study. Excluded from the analysis were patients who could not answer the questionnaire adequately either due to a decreased mental alertness or decreased cognitive function (cognitive disorders, sedated patients, Alzheimer’s disease, etc.). Four physicians and two clinical pharmacists received thorough training before the beginning of the study to ensure homogeneity and standardization of the data collection. Each physician counselled each patient about the warfarin treatment during the clinic visit; another counselling session was done by the clinical pharmacist, who was responsible of counselling the patient about the side effects of warfarin and the importance of patient’s compliance as well. A two-page brochure entitled “Warfarin: Understanding Side Effects and the Importance of Compliance” was prepared and distributed by the clinical pharmacist at the end of the face-to-face session. To assess patient’s adherence to treatment, we used the Medication Possession Ratio (MPR), defined as the proportion (or percentage) of days’ supply obtained over either refill interval, where last refill is the end point, or fixed refill, where a specific time period is set [20].

### Sample size calculation

A sample of 203 patients was targeted to allow for adequate power for bivariable and multivariable analyses to be carried out according to the Epi info sample size calculations with a population size of 4 million in Lebanon, a 15.7% expected frequency of knowledge about warfarin treatment [21], a 5% confidence limits [22]. We decided to distribute 350 questionnaires to take refusals into account.

### The questionnaire

The first part of the questionnaire aimed at collecting the patient’s demographic data (age, gender). The second part included a 17-item survey to measure patient’s knowledge about warfarin. This survey was designed by two clinical pharmacists based on existing documents that took into account education and counselling of warfarin therapy provided to patients during their hospital discharge (Additional file 1) [23–25]. An INR between 2 and 3 for all indications except for mitral valve replacement (INR range: 2.5-3.5) was considered as an adequate anticoagulation control [26]. The duration of warfarin therapy since the patient started the treatment was divided into 4 categories (2-3 months, 4-6 months, 7-12 months and more than a year) [2].

Furthermore, we assessed patients’ knowledge about warfarin and INR level recording (baseline) before the clinical pharmacist/physician counselling. As part of the effective anticoagulation management, frequent INR monitoring, dose alteration, and patient’s education are also necessary. Therefore, a health care professional (clinical pharmacist or physician) provided an oral counselling session for the patients to raise their level of awareness after the baseline assessment. Warfarin has a long half-life; following a single dose, the terminal elimination half-life is about 1 week, with a mean effective half-life of 40 h [27]. Individual verbal and written counselling was provided to each participant. Therefore, a second evaluation was scheduled 2 months after the counselling session to allow the drug to reach a steady-state concentration and determine whether patient’s knowledge had improved. Patients were divided into two different groups representing warfarin awareness and therapeutic INR status before and after the counselling session.

The survey included questions about drug information and dose, toxicity, drug-drug and drug-food interactions, and INR knowledge. For each question, every correct answer was given one mark then all scores were added. The final score in each section was calculated by adding the marks scored. The greater the score, the greater the level of knowledge.

### Data entry and analysis

Data entry was performed by one inspector who was not involved in the data collection process. Descriptive statistics were calculated for all study variables. This includes the mean and standard deviation for continuous measures, counts, and percentages for categorical variables. Paired t-tests were used to look for the difference between the patients’ knowledge before and after the counselling. The statistical package SPSS version 23 was used for all statistical analysis. Statistical significance was set at *p* < 0.05.

## Results

**Fig. 1:**
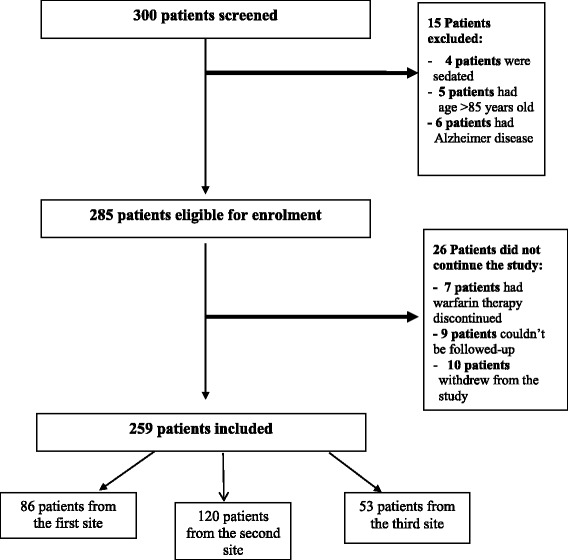
Flow chart of patient’s randomization and follow up

A total of 300 patients were screened. Consequently, 285 participants were enrolled in the study. All enrolled patients completed the warfarin awareness questionnaire before and after counseling whereas 259 completed the INR level assessment: 9 patients could not be contacted for the follow-up evaluation, 7 patients had their warfarin therapy discontinued by their doctors and 10 patients withdrew from the study. The randomization and follow-up of patients in this study are presented in Fig. 1. It is worth noting that all patients were adherent to their treatment throughout the study period and refilled their prescription without any delays.

Enrolled patients had a mean age of 61.18 ± 16.64 years; 53% were males. The majority of warfarin users had a past medical history of cardiovascular disease (62.5%) and hypertension (57.5%). Other patients’ characteristics are listed in Table 1.

**Table 1:** Demographic, social habits and other characteristics of patients treated with warfarin

Characteristic	Patient number (%)
Gender	
Male	151 (53)
Female	134 (47)
Age (years)	
Mean	61.18
Range	18-85
Smoking (Yes)	94 (33)
Alcohol (Yes)	13 (4.6)
Allergy (Yes)	13 (4.6)
Past Medical History^a^	
Previously healthy	11 (3.9)
Hypertension	164 (57.5)
Diabetes melllitus	103 (36)
Dyslipidemia	59 (20.7)
Cardiovascular disease	178 (62.5)

^a^Patient could respond to more than one option

The most frequently dispensed warfarin therapy indication was atrial fibrillation (47.7%), followed by VTE (31.6%), and valve disease/replacement (14.7%). In addition, 43.5% of the patients were taking warfarin for 2-3 months, 12.3% between 4 and 6 months 18.2% between 7 and 12 months and 26% for more than a year (Table 2).

**Table 2:** Indication and duration of warfarin therapy

Characteristic	Patient number (%)
Warfarin Therapy Indication^a^	
Atrial Fibrillation	136 (47.7)
Venous Thromboembolism	90 (31.6)
Valve disease/replacement	42 (14.7)
Stroke	13 (4.6)
Left Ventricular Dysfunction	4 (1.4)
Warfarin Therapy Duration	
2-3 months	124 (43.5)
4-6 months	35 (12.3)
7-12 months	52 (18.2)
> 1 year	74 (26)

^a^Patient could respond to more than one option

### Confirmatory factor analysis

We ran a confirmatory factor analysis on the whole sample (*n* = 300) to check the validity of the created questionnaire. The items converged over a solution of two factors that had an Eigen value over 1. High Cronbach’s alphas were also found for the full scale (0.856), showing good reliability.

### Evaluation of patients’ level of warfarin knowledge

**Fig. 2:**
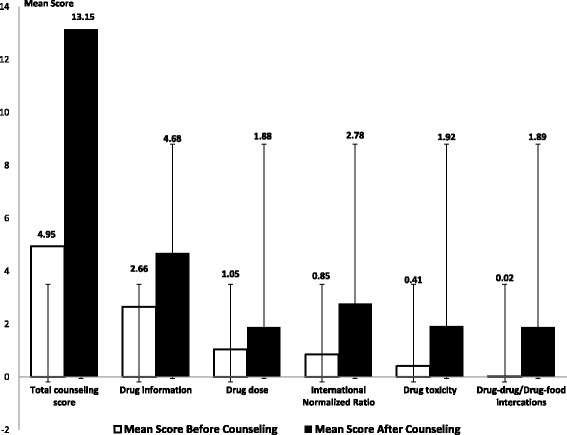
Comparison of the standard error and mean warfarin knowledge questionnaire score before and after counseling

Figure 2 shows the study findings relevant to the evaluation of the patient’s level of warfarin knowledge before and after the clinical pharmacist’s counselling. A higher mean knowledge about warfarin score was found after counselling as compared to before counselling (4.82 vs 13.2; *p* < 0.001). Likewise, the drug dose (1.05 vs 1.88), drug toxicity (0.41 vs 1.92), drug-drug and food-drug interactions (0.02 vs 1.89), therapeutic INR and general drug knowledge scores (2.66 vs 4.68) were significantly higher after as compared to before counselling (*p* < 0.001 for all variables).

From the total enrolled patients, a significantly lower percentage of patients with therapeutic INR values was found before counselling (37.2%, mean INR 1.69 ± 0.716), compared to those after counselling (74.4%, mean INR 2.11 ± 0.517) (*p* < 0.001) (Tables 3, 4 and 5).

**Table 3:** Number of therapeutic International Normalized Ratio level before and after counseling

Warfarin indication	Therapeutic INR level before counselling	Therapeutic INR level after counselling	*p*-value
Atrial Fibrillation	64/136 (47.1)	97/136 (71.3)	< 0.0001
Deep Venous Thrombosis	24/64 (37.5)	42/64 (65.6)	< 0.0001
Stroke	4/13 (30.8)	9/13 (69.2)	< 0.0001
Pulmonary Embolism	7/26 (26.9)	22/26 (84.6)	< 0.0001
Valve disease/replacement	7/42 (16.7)	38/42 (90.5)	< 0.0001
Systolic Left Ventricular Dysfunction	0/4 (0)	4/4 (100)	< 0.0001
Total	106/285 (37.2)	212/285 (74.4)	< 0.0001

For each warfarin indication, the number of patients who have reached the target INR level is illustrated above

Data are shown as number of patients over total with percentages in brackets

**Table 4:** International Normalized Ratio mean scores for patients treated with warfarin

	Number of patients	Mean ± Standard Deviation	*P*-value^a^
Before counseling	285	1.69 ± 0.716	< 0.001
After counseling^b^	259	2.11 ± 0.517	< 0.001

^a^Significant *p*-value < 0.001

^b^26 out of 285 patients were excluded: 7 females, 4 lost follow-up and 3 others discontinued warfarin; 19 males, 10 withdrew from the study, 4 discontinued warfarin and 5 others lost follow-up

**Table 5:** Paired t-test comparing means of patient’s knowledge questionnaire scores before and after counseling

Paired Samples Statistics					
		Mean	N	Std. Deviation	Std. Error
Pair 1	Patient knowledge after counseling/14	13.2	130	.000	.000
	Patient knowledge before counseling/14	4.82	130	2.600	.228
Pair 2	Patient knowledge about drug information after counseling/5	4.68	130	.000	.000
	Patient knowledge about drug information before counseling/5	2.66	130	1.097	.096
Pair 3	Patient knowledge about drug dose after counseling/2	1.88	130	.000	.000
	Patient knowledge about drug dose before counseling/2	1.05	130	.487	.043
Pair 4	Patient knowledge about INR after counseling/3	3.00	130	.000	.000
	Patient knowledge about INR before counseling/3	.85	130	1.138	.100
Pair 5	Patient knowledge about drug toxicity after counseling/2	1.92	130	.000	.000
	Patient knowledge about drug toxicity before counseling/2	.41	130	.679	.060
Pair 6	Patient knowledge about drug-drug and drug-food interactions after counseling/2	1.89	130	.000	.000
	Patient knowledge about drug-drug and drug-food interactions before counseling/2	.02	130	.175	.015

## Discussion

This is the first study that investigates the Lebanese patients’ knowledge concerning warfarin therapy on anticoagulation control (INR) within the Lebanese population. A higher percentage of our patients (74.4%) achieved good INR control after counselling compared to previous studies [2, 28].

Conflicting results have been reported in the literature regarding the relationship between warfarin knowledge and anticoagulation control. Our results are in agreement with previous findings [15, 29], but opposite to other findings [16]. In this study, patients completed the warfarin knowledge questionnaire on two scheduled follow-ups during the study, before and after the health care professional’s (HCP) counselling. Any question answered incorrectly was reinforced and the correct answers were documented. This was displayed by the mean score of the total warfarin knowledge which revealed a significant improvement after counselling, emphasizing the importance of the HCP implication in the process of disease prevention and treatment through adequate education and proper communication with patients. Our results support the findings of previous researches [30, 31] that showed that inadequate patient’s education and resultant misuse of warfarin correlate with increased difficulty in therapeutic outcome achievements. Other studies showed that patients’ knowledge and awareness about warfarin therapy significantly improved with effective education programs given by health care professionals, [32, 33].

Therefore, HCP, clinical pharmacists more importantly, can improve the patient’s quality of life and enhance his safety by reducing medication errors through the optimization of the appropriate dose, duration of the medication and encouraging routine monitoring.

Education can improve patients’ understanding of warfarin therapy and factors which affect INR control [1]. It is recommended that future studies examine such effect on the population. This suggests that following warfarin education and patient’s follow-up, a large number were able to manage, comply and monitor their warfarin medication, which in turn resulted in better therapeutic control. Previous studies have shown that adherence to warfarin therapy is significantly associated with improved anticoagulation (INR) control [15, 16]. In the present study, patients were educated about their warfarin therapy and taught about how to adjust doses according to their INR results and be compliant with their treatment. The findings showed that the percentage of patients achieving therapeutic INR values increased as the patient’s knowledge enhanced, in line with the findings of Collins et al. that showed that the positive effects of counselling and increasing patients’ warfarin knowledge were beneficial regardless of health literacy level [19]. Thus, HCP interventions significantly contributed to lower non-therapeutic INR scores.

### Limitations

Our study has some limitations: the questionnaire used was not validated. Patients were recruited from limited areas of Lebanon; thus, our results couldn’t be generalized to the entire population. In addition, lost to follow-up some patients might cause a selection bias. Our study did not assess patient’s knowledge about warfarin side effects both before and after counselling. When two categories of health care professionals are involved in the counselling, we cannot ascertain whose counselling is really effective or better than the other. Surely, it also makes differences how long someone is on warfarin, as those who are recently beginning the warfarin treatment will have a much greater likelihood of being out of range in the first month. Our study has elucidated a preliminary plan for a good therapeutic INR control in patients on warfarin, however, future studies are needed to address in depth these issues and evaluate the impact of long term follow-up has on patient’s warfarin knowledge, management, therapeutic outcomes, and complications.

## Conclusion

Warfarin therapy remains a potentially high risk and problematic challenge for both patients and health care professionals. The study results implied that educated patients about warfarin enhanced the knowledge about factors affecting therapeutic outcomes, and improved medication safety. Hence, HCPs have an important role in improving anticoagulation outcomes through a structured patient counselling and education.

## Additional file

Additional file 1: Patient’s knowledge evaluation questionnaire. Questionnaire we used during the data collection. (DOCX 28 kb)

## Abbreviations

A.Fib: Atrial fibrillation
HCP: Health care professional
INR: International Normalized Ratio
MPR: Medication possession ratio
SLVD: Systolic left ventricular dysfunction
SPSS: Statistical package for the social sciences
VTE: Venous thromboembolism
