# Supplementary material for: A multicenter, prospective study evaluating the impact of the clinical pharmacist-physician counselling on warfarin therapy management in Lebanon
Source: BMC Health Serv Res. 2018 Feb 1;18:80. doi: 10.1186/s12913-018-2874-7 (PMC5796596; doi:10.1186/s12913-018-2874-7)
Supplement: Additional file 1: — Patient’s knowledge evaluation questionnaire. Questionnaire we used during the data collection. (DOCX 28 kb) [file 12913_2018_2874_MOESM1_ESM.docx]

**Appendix 1.** Patient's knowledge evaluation questionnaire

1. What is the name of your prescribed VKA drug?  Knows  Doesn't know

2. What is the indication for your treatment?  Knows  Doesn't know

3. How long have you been taking this treatment?  Knows  Doesn't know

4. Did you already receive some information on this medicine?  Yes  No

Who gave you the information?  Physician  Pharmacist

 Nurse  Biologist

 Dentist  Other

What type of information?

5. Do you own a VKA monitoring handbook?  Yes  No

If yes, did you already read it?  Yes  No

6. When during the day do you take your medicine?  Knows  Doesn't know

7. Do you know what to do if you forget to take your medicine?  Knows  Doesn't know

8. Do you know what your medication INR level is?  Knows  Doesn't know

9. Do you know what the target INR is?  Knows  Doesn't know

10. Do you know the frequency of INR monitoring?  Knows  Doesn't know

11. What are the risks of taking a low dose?  Knows  Doesn't know

12. Do you know what the clinical symptoms in case of overdose are?

 Knows  Doesn't know

13. Are you aware of possible food interactions with your treatment?

 Knows  Doesn't know

14. Are you aware of what to watch for?  Knows  Doesn't know

15. Are you aware of possible drug interactions with your treatment?

 Knows  Doesn't know

16. Are you aware of what to watch for?  Knows  Doesn't know

17. What should you do if you would like to get pregnant?  Knows  Doesn't know

The questionnaire was designed by a pharmacist in partnership with a physical medicine and rehabilitation (PM&R) medical team based on existing documents. It included some elements from the VKA monitoring handbook published by the French Federation of Cardiology.^15-17^

VKA: vitamin k antagonists; INR: international normalized ratio
